# Understanding the Distribution of Muscidae Flies and Their Role as Vectors of Bacterial Pathogens in South Africa: A Review Using BOLD Barcoding Data

**DOI:** 10.1002/vms3.70934

**Published:** 2026-04-09

**Authors:** Veronica Phetla, Monica Mwale, Oriel Thekisoe, Maropeng Charles Monyama

**Affiliations:** ^1^ Department of Life and Consumer Sciences College of Agriculture and Environmental Science University of South Africa Pretoria South Africa; ^2^ Foundational Biodiversity Science South African National Biodiversity Institute Pretoria South Africa; ^3^ Unit for Environmental Sciences and Management North‐West University Potchefstroom South Africa

**Keywords:** DNA barcoding, diseases, South Africa

## Abstract

The role of Muscidae flies as vectors of bacterial pathogens with veterinary and public health significance necessitates accurate species identification and distribution mapping. This review analysed records from the Barcode of Life Data Systems (BOLD) and the South African National Biodiversity Institute (SANBI) national Diptera checklist, the BOLD Arthropoda progress report and literature, to assess South African Muscidae diversity, distribution and pathogen transmission. Through comparative analysis, we documented a total of 276 Muscidae fly species within the South African region. Three genera (*n* = 3) of the 38 genera, namely *Coenosia*, *Lispe* and *Musca*, demonstrated notable barcoding percentage gaps of 99%, 98% and 98%, respectively, indicating substantial genetic identification challenges. For the remaining 35 genera, a complete absence of barcode information was recorded, representing a 100% barcoding data gap. The striking disparity in Barcode Index Number (BIN) records across the three predominant fly genera (*Coenosia, Lispe* and *Musca*) reflects a concerning pattern in South African dipteran molecular taxonomy. The species *Musca domestica* demonstrated the most extensive pathogen diversity, carrying 22 distinct bacterial species, which include the ESKAPE group of pathogens. The results reveal critical gaps in taxonomic data alongside substantial pathogen transmission risks, necessitating integrated molecular‐taxonomic approaches and targeted disease control strategies.

## Graphical abstract

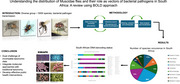

## 1 Introduction

The family Muscidae (Insecta: Diptera) exhibits remarkable diversity, comprising approximately 200 genera and 5000 species (Moon 2019; Sarwar 2020; Jana et al. 2025). This family comprises a taxonomically and ecologically diverse clade of calyptrate flies that function as key bioindicators, decomposers, pollinators and disease vectors across both anthropogenic and natural habitats (Marquardt 2004; Shaffer et al. 2007; Courtney et al. 2017; Kutty et al. 2019). The flies have great diversity in tropical and subtropical locations, where they can be found in almost every terrestrial habitat, from arctic tundras to tropical rainforests (Kirk‐Spriggs and Muller 2017; Skevington and Dang 2002). These flies can be classified into several different groups according to their preferred habitats and feeding habits (Greenberg 2019). Notable species within this family, including *Muscina stabulans* (false stable fly) (Fallén 1817), *Musca domestica* (house fly) (Linnnaues 1758), *Pollenia rudis* (cluster fly) (Fabricius 1794) and *Stomoxys calcitrans* (stable fly) (Linnnaues 1758), have been implicated in various public health and veterinary concerns as they are vectors of pathogens (Patra et al. 2018; Khamesipour et al. 2018; Moon 2019; Levchenko and Silivanova 2020; Khater et al. 2024).

In South Africa, muscid fly fauna is particularly abundant in the region (Moon 2019), where over 300 species have been discovered in a variety of habitats, including the savanna and the fynbos biome (Chilo 2019). Numerous endemic muscid species are the product of substantial diversification fostered by the varied temperature zones and biological niches of Southern Africa (Collen et al. 2008). South African muscids exhibit notable morphological and ecological diversity, including species that serve as specialized pollinators for specific local plants and others that act as decomposers facilitating nutrient cycling (Courtney et al. 2017; Chakraborti and Venkataraman 2018). The discovery of new muscid species within the boundaries by ongoing studies emphasizes the area's significance as a hub for muscid diversity in Africa (Couri et al. 2012). Researchers and health authorities have long acknowledged the global importance of dipteran insects in disease transmission, ecological function and public health (Wellman, 1950; Courtney et al. 2017; Verma et al. 2023). Studies have shown that these synanthropic flies can carry both susceptible and antibiotic‐resistant strains of bacteria, posing a significant threat to public health (Ercan et al. 2024). The presence of these flies in diverse transformed human habitats, such as farms and urban areas, underscores their importance and highlights the need for effective control measures to mitigate their impact (Boucheikhchoukh et al. 2019; Fukuda et al. 2020). Accurate identification and classification of fly species is increasingly important for biodiversity conservation and research (Potamitis 2014; Courtney et al. 2017). However, South Africa faces significant taxonomic challenges (Smith et al. 2008): comprehensive surveys of higher‐level insect groups remain incomplete, the taxonomic status of numerous fly species is uncertain, phylogenetic relationships among many taxa are poorly resolved, and there is a shortage of trained taxonomists specializing in Diptera. These gaps in knowledge hinder effective biodiversity management in one of the world's most species‐rich region (Boucheikhchoukh et al. 2019).

The study of fly taxonomy has undergone a significant change throughout time. Taxonomy studies initially started with basic microscopic tools in the 18th century when Linnaeus first documented *M. domestica* morphological characters. Improved compound microscopes and scanning electron microscopy (SEM) in the 19th and 20th centuries made it possible to examine external traits and ultrastructural features in great detail (Cortinhas et al. 2020). These tools served as the foundation for taxonomic keys for African muscids that were created by specialists like Zumpt 1950. Molecular techniques emerged in the 1980s and still being used to dates, initially utilizing mitochondrial genes (cytochrome c oxidase subunit I [COI], cytochrome b [cytB] and 16S ribosomal RNA [16S rRNA] and nuclear markers [18S, 28S rRNA, internal transcribed spacer ITS] regions) for species delimitation (Kocher et al. 1989; Renaud et al. 2012; Rotty et al. 2018). DNA barcoding standardized around the 5′ region of the mitochondrial DNA (mtDNA) COI gene in the early 2000s, though contemporary research has expanded to multi‐marker approaches (Achint and Singh 2021). More recently, studies have combined mtDNA with nuclear genes (CAD, EF‐1α, Period), and have also used next‐generation sequencing of whole mitochondrial genomes and broader genomic typing to support taxonomic studies (Achint and Singh 2021). These advanced techniques have proven essential for South African muscid studies, where cryptic species complexes often cannot be distinguished through morphology alone.

DNA barcoding of Muscidae flies in South Africa has a relatively recent history but has developed into an important area of research for taxonomy, biodiversity assessment and pest management (Kulenkampff 2019). Early efforts to DNA barcode flies began in the mid‐2000s, following the establishment of the Barcode of Life Data Systems (BOLD; [https://v3.boldsystems.org/] and the widespread adoption of COI as the standard marker for animal DNA barcoding globally using a 658 base‐pair segment (Shearer et al. 2002; Hebert et al. 2003). This method is widely adopted due to its effectiveness in distinguishing between species, especially when morphological identification is challenging and for taxon groups that have limited taxonomic expertise (Hebert et al. 2003; Ren et al. 2018; Achint and Singh; 2021). Data analysis and exchange through the BOLD BIN (Barcode Index Number) system are made easier, which acts as a centralized database for barcode sequences (Ratnasingham and Herbert 2007) is possible as clades define species. At present, the number of insect species records in regions like southern Africa has over 44,000 species spread across 7750 genera, 569 families and 25 orders (Myburgh et al. 2021). In South Africa, particularly in the Western Cape province, DNA barcoding has been applied to forensically important flies, including Muscidae, using the COI and ITS2 gene regions to successfully identify species, demonstrating sufficient discriminatory power (Cooke et al. 2018). The COI gene is favoured for its high interspecific divergence and ability to separate species in phylogenetic analyses (Ren et al. 2018; Achint and Singh 2021). Although DNA barcoding has revolutionized biodiversity research over the last 20 years by enabling rapid, accurate species identification and facilitating the discovery of cryptic species (Skevington and Dang 2002; Ivorra et al. 2021), little is known about the ecological and epidemiological roles these flies play as mechanical vectors in pathogen transmission particularly their capacity to carry and disseminate pathogenic bacteria between hosts, environmental reservoirs and human settlements remain poorly understood in the South African context (Baldacchino et al. 2013; Monyama et al. 2023).

Considering that South Africa is a biodiversity hotspot with over 244 fly species (Armstrong 2002) currently listed with most of these names requiring revision (https://hdl.handle.net/20.500.12143/9296) there is a need to support taxonomic capacity using advanced molecular techniques and verified curated databases such as BOLD (Stewart et al. 2024). Through the BOLD database, the establishment of reference libraries using DNA barcoding should help address the issue of ‘dark taxa’ and further promote international collaboration (Price et al. 2020). Therefore, this study aimed to synthesize and evaluate existing data regarding the progress of DNA barcoding of Muscidae flies from South Africa on the BOLD database and highlight geographical distribution patterns of Muscidae flies and their capacity as vectors of bacterial pathogen in South Africa. This review seeks to identify gaps in growing the reference database to support taxonomic research by screening the available DNA barcoding records on BOLD. Understanding the diversity and the distribution patterns of muscid flies across South African regions will also assist us in understanding their ecology and epidemiology, concerning their potential as vectors of bacterial pathogens to inform decision‐making that will benefit conservation and public health programs for biodiversity management.

## 2 Materials and Methods

**FIGURE 1:**
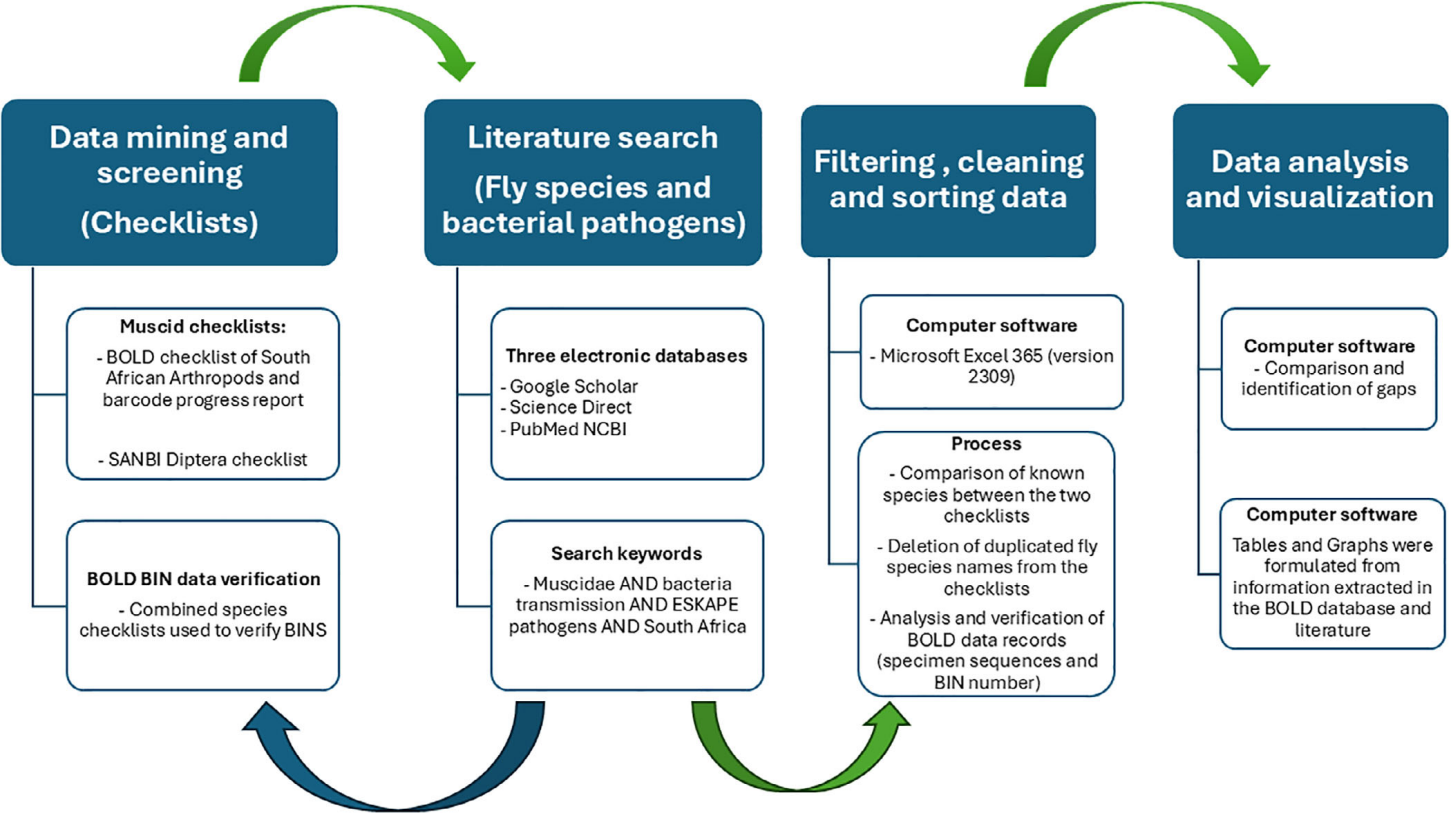
Workflow of the methodology steps for data screening, sorting, verification of data and data analysis.

Data was mined and downloaded from the BOLD database to obtain a progress report of barcoded insect species from South Africa using the BOLD checklist of South African Arthropods (BOLD checklist code: CL‐SAART South Africa‐ Arthropoda; updated 9 September 2019, https://bench.boldsystems.org/index.php/Checklists_Management/). The checklist included known species records with the taxonomic information on six taxonomic hierarchy levels: phylum, order, family, genus, species and subspecies. The BOLD checklist was refined to include DNA barcode data exclusively for Muscidae specimens from South Africa. The progress report provided additional information associated with the checklist on the number of available voucher specimen records of each species, specimens with COI records and specimens that have compliant DNA sequences (consisting of both the forward and reverse sequences trace files). In addition, a SANBI Diptera checklist (last updated in January 2024) was downloaded for comparison with the BOLD progress report (https://hdl.handle.net/20.500.12143/9296). Apart from taxonomic hierarchy, the SANBI checklist also includes species data on distribution (global and national). All the checklists including the progress report were filtered in Microsoft Excel (365, version 2309) for the family Muscidae (Diptera) before comparison. After filtering, the two checklists were merged to identify differences in number of known species. All species from both lists including new entries which were missing from either list were verified for taxonomic rank and name accuracy across all levels. The resulting records obtained from the combined datasets were further cleaned by removing data fields (phylum, class, order and family) considered irrelevant to this analysis. To identify gaps in the barcode records, the checklist was cross‐referenced against BOLD for each taxon group to determine the percentage of known species with records on the database (Figure 1).

Manual searches were done using the BOLD systems BIN database using the terms ‘Muscidae’ and ‘South Africa’ as keywords to further validate BIN records, as this data includes locality information for unpublished BOLD records that share BINS with South African records. This was conducted by extracting BINs from only verified South African localities. For each South African barcoded fly species, the following information was documented which included: the specimens with DNA sequences, verified barcode sequences and the number of species‐specific BINs. To assess the accuracy of taxonomic assignments based on sequence data, BIN discordances were assessed by considering BIN splits (species name with more than one BIN on BOLD) and BIN merges (more than one species name included in a BIN). BIN splits and merges highlight areas of taxonomic interest, as discordant BIN assignments may indicate inconclusive species delineation or hidden diversity. Furthermore, we estimated the number of specimen records without BINs or barcodes to identify potential datasets that might be available for processing (i.e., backlogs of data that require the capacity to deposit barcodes on BOLD) or to highlight issues with the barcoding process (Figure 1).

In addition, published peer‐reviewed articles from accredited journals explicitly reporting on the distribution of Muscidae flies and their role as vectors of bacterial pathogens in South Africa were identified and reviewed following the recommended standards for a scoping review (Munn et al. 2018). Three electronic databases, Google Scholar (https://scholar.google.com), Science Direct (https://www.sciencedirect.com/) and PubMed (http://www.ncbi.nlm.nih.gov/pubmed/), were used to search for relevant literature. The following keywords and Boolean operators (AND, OR) were used in the search: Muscidae AND bacteria transmission AND ESKAPE pathogens AND South Africa. Relevant articles were first identified by screening through their titles and abstracts. Articles eligible through the screen were then analysed. To determine representation percentages and data gaps, all generated data, including South African species distributions, specimens, barcode numbers, sequences and BINs were examined (Figure 1). The results were subsequently presented using graphs created using Microsoft Excel 365 (version 2309).

## 3 Results

### 3.1 Diversity and Distribution of Muscidae Flies in South Africa

Analysis of checklists as well as literature of South African species identified 276 unique Muscidae fly species names from 39 genera. The consolidated list of species could be an underestimate as the taxonomy of most South African species has not been verified with the number of species being understudies. This species list included 245 known species that were recorded on the combined checklists (BOLD and SANBI) as well as 31 additional species that were obtained from literature resources to consolidate the final checklist number of Muscidae fly species. The 31 species obtained from literature included seven newly described endemic species, namely *Coenosia curiosa* sp. nov., *Helina harrisorum* sp. nov, *Hydrotaea tantula* sp. nov., *Limnophora antennalis* sp. nov., *L. diminuta* sp. nov., *S. bella* sp. nov. and *S. brunnea* sp. nov. (Table S1). In terms of the combined checklist, seventeen (*n* = 17) SANBI national checklist species were missing from the BOLD database, while four species records on BOLD were not on the SANBI National checklist (Table S1).

The geographical distribution analysis of the 276 fly species revealed the widespread occurrence across all nine provinces of South Africa, demonstrating a comprehensive spatial coverage of this fly family throughout the country's diverse ecological regions with KwaZulu Natal province displaying the highest diversity of fly species, followed by Limpopo, Mpumalanga, Gauteng, Eastern Cape, Free State, Western Cape, Northern Cape and lastly North West (TableS1). Analysis of provincial distribution patterns revealed varying occurrence rates among the Muscidae genera across South Africa. Nine genera namely *Anaphalantus, Atherigona, Coenosia, Dichaetomyla, Helina, Limnophora, Lispe, Musca* and *Stomoxys*, showed extensive distribution, occurring in five to nine provinces (Table S1). Moderate distribution was observed in 15 genera, present in three to four provinces, while 12 genera demonstrated limited distribution, occurring in only one to two provinces. Overall distribution per genera revealed that two (*Hebecnema* and *Ophyra*) lacked specific locality data within South Africa (Table S1), underscoring the complexity of fly species distribution and the potential need for further ecological research to comprehensively map their presence across the country's diverse ecological regions.

**FIGURE 2:**
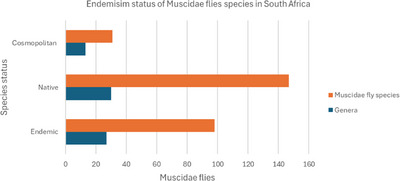
Bar graph representing fly species in the South African checklist categorized according to their native status.

This study delineates the occurrence and endemic status of Muscidae fly species through comprehensive taxonomic analysis, offering a detailed portrait of South Africa's fly fauna. Of the total 276 fly species, 35.5% (*n* = 98) are endemic to South Africa, 53.3% (*n* = 147) are native and 11.2% (*n* = 31) have a cosmopolitan endemism species status (Table S1, Figure 2). These results suggest a diverse fly fauna in South Africa, with more than half being native species, a significant portion being endemic, and a small percentage having a global distribution.

### 3.2 Barcode Data Gaps of Muscidae Flies in South Africa

**FIGURE 3:**
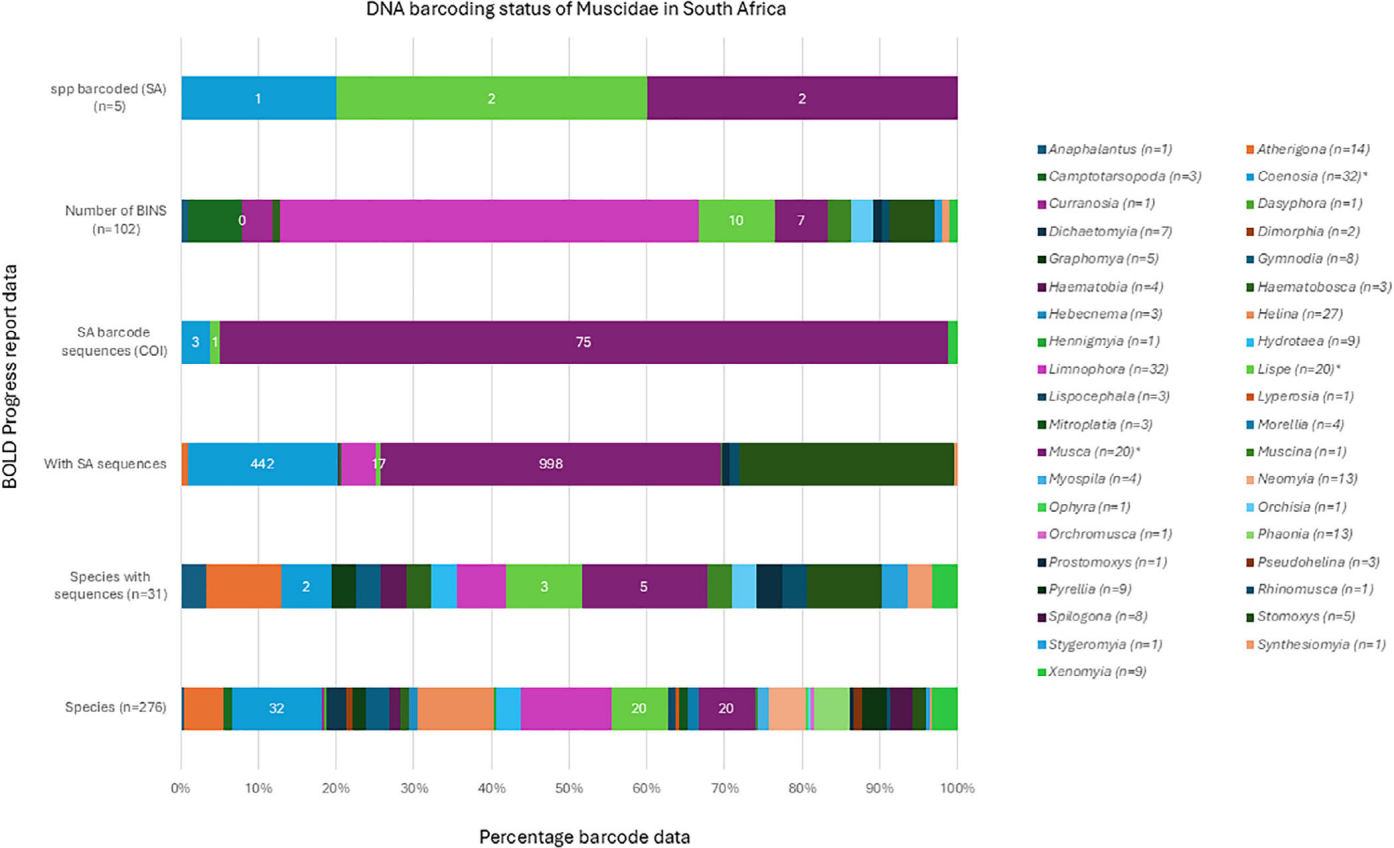
DNA barcoding status of South African Muscidae by genus, based on six BOLD Progress Report metrics, including total species richness, COI sequence availability, BIN assignments, and barcoding completeness within South Africa.

The Barcode of Life Data (BOLD) database was used to examine and verify the taxonomic records of fly species currently listed in South African entomological checklists, offering a molecular‐based approach to species identification and confirmation. Globally, these species encompassed 1675 total barcodes, with a mere 80 barcodes recorded within South Africa (Table S2, Figure 3). Since there were more BINs than barcoded species, there may have been genetic splits and a higher chance of misidentification, suggesting that the 102 BINs linked to these records indicate substantial *COI* genetic diversity.

There are three genera that have the highest barcoding records appearing on the BOLD database. These genera included *Coenosia, Lispe* and *Musca* (Table S2, Figure 3). The genetic analysis of the genus *Coenosia* flies in South Africa revealed significant limitations in current taxonomic documentation. Out of a comprehensive collection of 442 genetic sequences representing 32 known species, only three sequences were specifically attributed to South African specimens. This highlights a notable scarcity of genetic characterization for this fly genus within the region. With merely one species barcoded in South Africa and an absence of BIN records, the study underscores substantial gaps in understanding the genetic diversity and taxonomic composition of *Coenosia* flies (Figure 3). Secondly, the genetic analysis of the *Lispe* genus also revealed complex and limited genetic documentation within South Africa's fly species databases. Out of the total 276 fly species reported, 20 fly species belong to the *Lispe* genus, with only three species having genetic sequences available. Notably, 17 species were associated with South African sequences, yet merely one specimen possessed a definitive South African barcode. The records in the databases further uncovered 10 BIN records, of which only two were linked to South African barcodes. The discrepancy between species count, sequence availability and barcode representation indicates the need for more comprehensive molecular taxonomic studies to fully understand the genetic diversity and distribution of *Lispe* flies in the region (Figure 3). Lastly, the genus ‘*Musca*’ database revealed considerable gaps in taxonomic and genetic documentation. Of the 1529 recognized fly species within this genus recognized on the BOLD system, only 20 species (7.2%) had any recorded entries in the database. Genetic sequencing was even more limited, with only five species having molecular data. While 998 specimens collected in South Africa contained sequence information, these represented just 75 distinct barcode records from South African specimens. The molecular diversity was further categorized into seven unique BINs (Figure 3). Notably, only two *Musca* species have been formally barcoded from South African populations, highlighting a significant taxonomic impediment for this medically and economically important dipteran genus in the region. This pronounced discrepancy between the total number of recognized species and their genetic characterization in South Africa underscores an urgent need for comprehensive molecular taxonomic studies to address these knowledge gaps, enhance species identification capabilities and support effective biodiversity conservation and public health management strategies within the region.

Bacterial pathogen results showed a significant association between Muscidae fly species and diverse bacterial pathogens transmitted by these flies, highlighting and alerting their potential key players as disease vectors. A total of 9 fly species have been identified to be bacterial transmitters of 38 bacterial pathogens that are of veterinary and health importance (Table S3, Table S1A), of which some are classified as critical pathogens according to the World Health Organisation (WHO). The fly species with the highest bacterial transmission is *M. domestica*, and it acts as a reservoir for transmitting resistance factors. This fly demonstrated the most extensive pathogen diversity, carrying 22 distinct bacterial species which include WHO's critical listed bacteria pathogens belonging to the ESKAPE group (*Enterococcus faecium*, *Staphylococcus aureus, Klebsiella pneumoniae, Acinetobacter baumannii, Pseudomonas aeruginosa, Enterobacter spp., Escherichia coli), Salmonella spp*. and notable bacilli causing typhoid, cholera, pneumonia and anthrax (Table S3). This was followed by *M. sorbens* transmitting a total of 10 bacterial pathogens (*Anthrax bacillus, Campylobacter jejuni, Chlamydia trachomatis, Coxiella burnetii, E. coli, Mycobacterium leprae, M. tuberculosis, P. aeruginosa, S. aureus* and *Treponema pertenue*). Thirdly, *M. autumnalis*, which resulted in the third fly species with the most bacterial pathogen transmissions, with a total of nine bacterial pathogens being transmitted by the fly. These pathogens include *C. jejuni, Entamoeba coli, E. faecium, E. coli, K. pneumoniae, Moraxella bovis, P. aeruginosa, S. aureus* and *Streptococcus equi* (Table S1A, S2).

Fly species with the least bacteria transmission are *Haematobia spinigera* and *Haematobosca uniseriata* transmitted the least bacteria and were both linked to *M. bovis*, suggesting a specific veterinary health concern.

## 4 Discussion

Research continues to highlight considerable gaps in the current taxonomic data of Muscidae in South Africa. The comparative examination of the distribution of Muscidae species in South Africa additionally demonstrates noteworthy patterns in biodiversity documentation. The substantial overlap of 224 fly species between the SANBI national checklist and the BOLD system demonstrates a robust foundation for muscid taxonomy in South Africa. The extent of data comparison is consistent with previous study research in other insect and arachnids areas, including studies by Whittington (2018) and Khumalo et al. (2024), who discovered similar amounts of database overlap in listings of Arthropoda databases/checklists. The addition of 31 species through literature review and other checklists underscores the critical importance of incorporating historical taxonomic work into modern molecular databases, a point noted by Whittington (2018) in their comprehensive review of Afrotropical Diptera. The discovery of five species in BOLD not previously recorded in the SANBI checklist (*Dasyphora affricana, Lispe andrewi, L. hirtibasis, L. spinipes* and *S. trisetalis*) represents significant additions to the known South African muscid fauna. This discovery matches with a recent research study by Zielke (2019)), who found comparable database discrepancy patterns in Polish muscid collections. Conversely, the absence of four species from BOLD that are present in the SANBI national Diptera checklist (*L. trisetalis, Pyrellina affricana, S. spinipes* and *Xenomyia hirtibasis*) may indicate either taxonomic uncertainty, misidentification or sampling gaps, which is a challenge noted by Stewart et al. (2024) in their analysis of South African insect diversity on BOLD, where they found that despite Diptera's good representation on BOLD (121,513 records), only 77 of approximately 150 families known from southern Africa were represented, indicating considerable under sampling. Particularly noteworthy is the identification of species absent from either of the checklists, especially given that several of these species, such as *Stomoxys calcitrans*, are known to be common and widely distributed (Greenberg 1973; Roy and Dasgupta 1975; Briggs and Milligan 1977; Cohen et al. 1991; Pont 1991; Sulaiman et al. 2000; Holt et al. 2007; Förster et al.; Bahrndorff et al. 2013; Khamesipour et al. 2018; Patra et al. 2018; Geden et al. 2021; Monyama et al. 2022; Yin et al. 2022; Monyama et al. 2023). This discrepancy likely reflects historical sampling biases and the challenges of maintaining updated taxonomic records. The absence of these well‐known species from both databases suggests a critical need for improved integration of historical and contemporary sampling efforts. In addition, more advanced taxonomic identification tools such as STEM and Stereomicroscopes, coupled with genetic characterization using polymerase chain reaction (PCR) for more accurate and robust results.

Significant regional heterogeneity in South Africa's muscid diversity can be observed in the province distribution patterns. This diverse range is consistent with more general trends in southern African insect biodiversity reported by (Mva et al. 2022). The influence of environmental conditions, especially patterns of rainfall and temperature, is probably indicated in the observed diversity gradients. Furthermore, habitat heterogeneity correlates with species richness across provinces, supporting Haslett (2001) results about the connection between muscid diversity and landscape complexity. Several sample challenges, such as historical sampling effort variances among provinces, accessibility of various regions, concentration of research institutions and focus of prior collection efforts, may have an impact on the observed distribution patterns. These potential biases align with observations by Courtney et al. (2017) and Mva et al. (2022) regarding the impact of sampling efforts on perceived insect diversity patterns in Africa. The final compilation of 276 fly species represents a significant update to the known muscid fauna of South Africa, with important implications for both taxonomic understanding and conservation planning.

The study additionally reveals several critical research gaps in understanding Muscidae fly distribution across South Africa, notably the complete absence of locality data for three genera (*Hebecnema, Lyperosia* and *Ophyra*), and significant variations in species diversity across provinces with North West showing the lowest representation. Methodological limitations are evident in the uneven geographical coverage, with 12 genera demonstrating extremely limited distribution across only one to two provinces, suggesting potential sampling biases or incomplete survey techniques. The research lacks explanatory insights into the ecological factors driving these distribution patterns, such as why certain fly genera are extensively spread while others are rare, and does not provide clear information about the sampling methods used. These gaps highlight substantial opportunities for future research to conduct more comprehensive surveys, particularly in underrepresented provinces, investigate the ecological mechanisms influencing fly distribution, develop more robust sampling strategies and focus on genera with minimal or no current locality information, ultimately aiming to create a more complete understanding of Muscidae fly biodiversity in South Africa.

DNA barcoding efforts in South Africa continue to face several notable gaps despite South Africa's exceptional biodiversity (Adamowicz et al. 2017). While significant progress has been made in species barcoding certain taxonomic groups, particularly among commercially important species, remain unbarcoded (Adamowicz et al. 2017). This is particularly notable in arid regions and within certain arthropod groups such as Muscidae flies. The comprehensive nature of this analysis provides a solid foundation and reason for upcoming research while highlighting and criticizing the ongoing challenges in documenting and monitoring insect biodiversity in South Africa. The integration of molecular and traditional taxonomic approaches, combined with systematic sampling efforts, will be of paramount importance for maintaining accurate and current species databases in this biodiversity‐rich region (Samways et al. 2010). This scoping review taxonomic investigation of Muscidae fly species in South Africa revealed profound genetic characterization challenges, with 38 genera displaying significant barcoding gaps. Three genera *Coenosia, Lispe* and *Musca* showed partial genetic documentation with barcoding percentage gaps of 99%, 98% and 98%, respectively, while the remaining 36 genera exhibited a complete 100% absence of barcode information. These findings underscore critical limitations in molecular taxonomic research, highlighting urgent needs for comprehensive genetic sampling, precise species identification, and expanded molecular systematics studies to address the substantial knowledge gaps in South African Muscidae fly biodiversity. The extensive barcoding deficiencies not only impede precise taxonomic classification but also restrict our understanding of these fly species' evolutionary relationships, ecological roles and potential biodiversity contributions in the region. Muscidae flies serve as significant mechanical vectors of various bacterial pathogens due to their synanthropic nature and feeding habits described by Hassan et al. (2021) and Stoffolano (2022). These flies can transfer bacteria through their regurgitation, defecation and external body surfaces, particularly their legs and proboscis, which are equipped with numerous setae that can harbour microorganisms, as also explained by Makhahlela (2019) and Tan et al. (2024).

The investigation into Muscidae fly species as bacterial vectors reveals a significant public health concern, with *M. domestica*, in the review analysis, emerging as the most critical pathogen carrier. By harbouring 22 distinct bacterial species, including *E. coli*, *Salmonella* spp., *S. aureus* and pathogens responsible for typhoid, cholera, pneumonia and anthrax, these flies demonstrate remarkable potential for disease transmission. Other species, like *M. sorbens* and *M. crassirostris* also contribute to this epidemiological risk, carrying bacteria such as *C. trachomatis, M. tuberculosis* and *Bacillus anthracis*. These findings underline the critical need for comprehensive vector control strategies and highlight the often‐overlooked role of flies as mechanical vectors in the environment. The scoping review suggests that understanding and mitigating the bacterial transmission capabilities of Muscidae species could significantly impact public health interventions, particularly in regions with limited sanitation infrastructure and, importantly veterinary settings such as zoo parks and farms. Further epidemiological research is crucial to fully comprehend the transmission dynamics, develop targeted control methods, and assess the full extent of potential disease spread facilitated by these ubiquitous insects. The scoping research provides an alarming insight into the potential of these fly species to carry and spread a diverse range of bacterial pathogens that pose serious risks to veterinary, and human health and some in agricultural settings (Gwenzi et al. 2021).

As *Musca domestica* emerges as the most concerning vector in this study, carrying 22 distinct bacterial species. The findings of the study showed that this fly species harboured all major ESKAPE pathogens, which are known for their antibiotic resistance and clinical treatment challenges. These include *E. faecium, S. aureus, K. pneumoniae, A. baumannii, P. aeruginosa, Enterobacter* spp. and *E. coli* (Karami et al. 2021). These findings correspond with many studies where the results were obtained (Monyama et al. 2023; De Wet et al. 2024). In addition, these findings underscore the critical public health significance of Muscidae flies as potential mechanical vectors for a wide range of bacterial infections, emphasizing the need for comprehensive vector control strategies and further epidemiological research.

South African barcoding gaps continue to suggest an urgent need for more extensive genetic research and molecular taxonomic studies focused on the *Coenosia* genus in South Africa. The limited genetic data indicates potential unexplored biodiversity and presents significant opportunities for future scientific investigation to comprehensively map and understand the genetic landscape of these fly species in the region. The low number of barcoded sequences not only reflects current research constraints but also points to the complex challenges in the genetic identification and classification of fly species within South African ecosystems. Such limitations emphasize the importance of continued molecular research to enhance our understanding of local entomological biodiversity. These findings highlight significant gaps in the genetic characterization of the *Lispe* genus in South Africa. The minimal genetic representation with just one specimen barcoded and limited genetic sequences suggests substantial opportunities for future research.

The study strongly suggests the urgent need for comprehensive vector control strategies and further epidemiological research. Recommendations include developing integrated pest management approaches, improving sanitation and waste management and creating targeted interventions for Muscidae fly populations. In addition, there is a critical need for continued research to understand the transmission dynamics, investigate geographical variations in pathogen distribution, and develop more effective methods of tracking bacterial transfer. Public health interventions must be multifaceted, focusing on raising awareness about fly‐borne bacterial transmission, promoting personal, animal and environmental hygiene and developing preventive medical protocols for all sectors.

## 5 Conclusion

The research shows significant taxonomic and DNA barcoding gaps in the South African Muscidae, with 38 genera showing substantial barcoding deficiencies. While there is a robust foundation of 224 species overlap between SANBI national Diptera checklist and BOLD databases, critical gaps remain in the databases, particularly in the genetic characterization of Muscidae fly species. The study also emphasizes the serious public health implications of Muscidae fly species as bacterial vectors in many settings, with *M. domestica* being identified as the most significant carrier, harbouring 22 distinct bacterial species including ESKAPE pathogens. This dual challenge of incomplete taxonomic data and significant disease transmission potential highlights the urgent need to integrate molecular approaches with traditional taxonomy while developing effective public health interventions.

## Author Contributions

**Veronica Phetla**: conceptualization (lead), methodology (lead), data curation (lead), formal analysis (lead), visualization (lead), writing – original draft (lead), writing – review and editing (lead). **Monica Mwale**: formal analysis (lead), visualization (lead), writing – review and editing (support). **Oriel Thekisoe**: conceptualization (support), writing – review and editing (support). **Charlse Monyama**: conceptualization (support), writing – review and editing (support).

## Funding

The authors have nothing to report.

## Ethics Statement

The authors confirm that the ethical policies of the journal, as noted on the journal's author guidelines page, have been adhered to.

## Conflicts of Interest

The authors declare no conflicts of interest.

## Supporting information




**Supporting File 1**: vms370934‐sup‐0001‐tableS1.xlsx.

**Supporting File 2**: vms370934‐sup‐0002‐tableS1.docx.

**Supporting File 3**: vms370934‐sup‐0003‐tableS2.xlsx.

**Supporting File 4**: vms370934‐sup‐0004‐tableS3.xlsx.

## Data Availability

Data generated or analysed during this study are provided in full within the published article and its Supporting Information.
