# Supplementary material for: Understanding the Distribution of Muscidae Flies and Their Role as Vectors of Bacterial Pathogens in South Africa: A Review Using BOLD Barcoding Data
Source: Vet Med Sci. 2026 Apr 9;12(3):e70934. doi: 10.1002/vms3.70934 (PMC13064580; doi:10.1002/vms3.70934)
Supplement: Supplementary file 2 — Supporting File 2: vms370934‐sup‐0002‐tableS1.docx. [file VMS3-12-e70934-s004.docx]

| **S1A Table: Bacterial pathogens transmitted by Muscidae fly species** | | |
| --- | --- | --- |
| **Muscidae fly species** | **Bacterial pathogens** | **References** |
| *Haematobia spinigera* | *Moraxella bovis* | Arends et al. 1982 |
|  |  |  |
| *Haematobosca uniseriata* | *Moraxella bovis* | Arends et al. 1982 |
|  |  |  |
| *Musca autumnalis* | *Moraxella bovis, Klebsiella spp., Escherichia coli, Pseudomonas aeruginosa, Campylobacter jejuni,  Enterococcus faecium, Klebsiella pneumoniae, Salmonella enterica, Staphylococcus aureus* | Burton, 1966; Briggs and Milligan, 1977; Blazar et al. 2011; Onwugamba et al. 2018 |
|  |  |  |
| *Musca confiscata* | *Escherichia coli, Salmonella spp., Staphylococcus aureus* | Pont, 1991 |
|  |  |  |
| *Musca crassirostris* | *Escherichia coli, Moraxella bovis, Staphylococcus aureus, Bacillus anthracis, Salmonella spp, Shigella spp, Anaplasma marginale* | Roy and Dasgupta, 1975; Pont, 1991; Desquesnes et al. 2019 |
|  |  |  |
| *Musca domestica* | *Klebsiella spp,  Escherichia coli, Aeromonas hydrophila, Entamoeba coli, Campylobacter jejuni, Enterococcus faecalis, Staphylococcus aureus, Shigella spp., Clostridium perfringens, Vibrio cholerae, Listeria monocytogenes, Yersinia enterocolitica, Edwardisella spp, Burkholderia pseudomalliei, Cholera bacillus, Paratyphoid bacillus, Typhoid bacillus, Pneumonia bacillus, Anthrax bacillus, Campylobacter spp., Salmonella spp., Pseudomonas aeruginosa* | Greenberg, 1973; Roy and Dasgupta, 1975; Briggs and Milligan, 1977; Cohen et al. 1991; Pont, 1991; Sulaiman et al. 2000; Holt et al. 2007; Förster et al. 2009; Bahrndorff et al. 2013; Khamesipour et al. 2018; Geden et al. 2021; Monyama et al. 2022; Olagunju, 2022; Yin et al. 2022; Monyama et al. 2023 |
|  |  |  |
| **Muscidae fly species** | **Bacterial pathogens** | **Refernces** |
| *Musca sorbens* | *Chlamydia trachomatis, Escherichia coli, Campylobacter jejuni, Staphylococcus aureus, Coxiella burnetii, Anthrax bacillus, Mycobacterium leprae, Mycobacterium tuberculosis, Treponema pertenue, Pseudomonas aeruginosa* | Roy and Dasgup, 1975; Robinson et al. 2024 |
|  |  |  |
| *Stomoxys calcitrans* | *Bacillus anthracis, Pasteurella multocida, Erysipelothrix rhusiopathiae, Francisella tularensis, Dermatophilus congolensis, Staphylococcus aureus,  Anaplasma marginale, Coxiella spp., Enterobacter sakazakii, Paratyphoid bacillus* | Schuberg and Boing, 1914; Wellman, 1950; Turell and Knudson, 1987; Richard and Pier, 1966; Patra et al. 2018; Baldacchino et al. 2013; Makhahlela et al. 2022 |
| *Synthesiomyia nudiseta* | *Entamoeba coli* | Ivorra et al. 2021 |
